# Evaluation of Riboflavin Transporters as Targets for Drug Delivery and Theranostics

**DOI:** 10.3389/fphar.2019.00079

**Published:** 2019-02-06

**Authors:** Lisa Bartmann, David Schumacher, Saskia von Stillfried, Marieke Sternkopf, Setareh Alampour-Rajabi, Marc A. M. J. van Zandvoort, Fabian Kiessling, Zhuojun Wu

**Affiliations:** ^1^Institute for Experimental Molecular Imaging, University Clinic, RWTH Aachen University, Aachen, Germany; ^2^Institute for Molecular Cardiovascular Research, University Clinic, RWTH Aachen University, Aachen, Germany; ^3^Institute of Pathology, University Clinic, RWTH Aachen University, Aachen, Germany; ^4^Department of Genetics and Molecular Cell Biology, School for Cardiovascular Diseases (CARIM), School for Oncology and Developmental Biology (GROW), Maastricht University, Maastricht, Netherlands

**Keywords:** riboflavin, RFVT, theranostic, nanocarrier, cancer therapy, targeting molecules

## Abstract

The retention and cellular internalization of drug delivery systems and theranostics for cancer therapy can be improved by targeting molecules. Since an increased uptake of riboflavin was reported for various cancers, riboflavin and its derivatives may be promising binding moieties to trigger internalization via the riboflavin transporters (RFVT) 1, 2, and 3. Riboflavin is a vitamin with pivotal role in energy metabolism and indispensable for cellular growth. In previous preclinical studies on mice, we showed the target-specific accumulation of riboflavin-functionalized nanocarriers in cancer cells. Although the uptake mechanism of riboflavin has been studied for over a decade, little is known about the riboflavin transporters and their expression on cancer cells, tumor stroma, and healthy tissues. Furthermore, evidence is lacking concerning the representativeness of the preclinical findings to the situation in humans. In this study, we investigated the expression pattern of riboflavin transporters in human squamous cell carcinoma (SCC), melanoma and luminal A breast cancer samples, as well as in healthy skin, breast, aorta, and kidney tissues. Low constitutive expression levels of RFVT1–3 were found on all healthy tissues, while RFVT2 and 3 were significantly overexpressed in melanoma, RFVT1 and 3 in luminal A breast cancer and RFVT1–3 in SCC. Correspondingly, the SCC cell line A431 was highly positive for all RFVTs, thus qualifying as suitable *in vitro* model. In contrast, activated endothelial cells (HUVEC) only presented with a strong expression of RFVT2, and HK2 kidney cells only with a low constitutive expression of RFVT1–3. Functional *in vitro* studies on A431 and HK2 cells using confocal microscopy showed that riboflavin uptake is mostly ATP dependent and primarily driven by endocytosis. Furthermore, riboflavin is partially trafficked to the mitochondria. Riboflavin uptake and trafficking was significantly higher in A431 than in healthy kidney cells. Thus, this manuscript supports the hypothesis that addressing the riboflavin internalization pathway may be highly valuable for tumor targeted drug delivery.

## Introduction

Sustaining proliferative signaling and reprogramming of energy metabolism are two key characteristics of tumorigenesis and tumor progression (Hanahan and Weinberg, 2011). Timely pharmacotherapies exploit markers for tumor targeted drug delivery that are, among others, related to enhanced angiogenesis (Zhao and Adjei, 2015), hypoxia (Wilson and Hay, 2011), proliferation (Feitelson et al., 2015), and metabolism (Luengo et al., 2017).

Recent studies shed light onto the metabolic role of riboflavin and its association with a wide array of diseases (Thakur et al., 2017), including cancer (Yang et al., 2013; Ozsvari et al., 2017). Riboflavin is an essential vitamin and versatile micronutrient involved in a spectrum of redox reactions through the cofactors flavin mononucleotide (FMN) and flavin adenine dinucleotide (FAD), which act as essential electron carriers during cellular respiration and energy production (Mansoorabadi et al., 2007). Unsurprisingly, studies have shown riboflavin to play an important role in tumor growth (Rao et al., 1999; Yang et al., 2013; Li et al., 2017). Not only did riboflavin supplementation increase cancer cell proliferation, invasion and migration (Yang et al., 2013), ultimately increasing tumor survivability, but the inhibition of flavin-containing enzymes also arrested tumor growth (Ozsvari et al., 2017). Furthermore, riboflavin and its derivatives have been successfully utilized as tumor targeting ligands *in vitro* and *in vivo*, showing enhanced tumor-specific accumulation of riboflavin-targeted nanotubes carrying paclitaxel in MCF-7 cells (Singh et al., 2016) as well as riboflavin-targeted liposomes in murine A431 and PC3 tumor xenografts (Tsvetkova et al., 2017). However, although preclinical studies using riboflavin targeting provided promising results, there is still a lack of mechanistic insights into the cellular uptake and trafficking. Furthermore, clinical validation of RFVT overexpression in cancer is still pending.

Until now, three riboflavin transporters, RFVT1, 2 and 3, have been identified (Yonezawa et al., 2008; Yamamoto et al., 2009; Yao et al., 2010; Yonezawa and Inui, 2013). The uptake function of the three RFVT isomers are highly specific toward native riboflavin (Jin et al., 2017), with Km values in the lower micromolar range (Yao et al., 2010). A systematic investigation of RFVT mRNA levels in healthy human tissue revealed distinctive tissue-specific RFVT expression profiles, suggesting RFVTs’ role in the regulation of cellular riboflavin homeostasis (Yao et al., 2010). However, the individual role of each RFVT remains unclear. Elevated levels of RFVT3 were observed in glioma and esophageal squamous cell carcinoma tissue (SCC) (Jiang et al., 2014; Fu et al., 2016), indicating RFVT3 to be mainly responsible for riboflavin uptake in tumors. Studies investigating the uptake mechanism of riboflavin have provided contradicting results. While some studies suggest riboflavin uptake to be dynamin-dependent and therefore driven via receptor-mediated endocytosis (Huang et al., 2003; Foraker et al., 2007; Bareford et al., 2008), other studies indicate a non-endocytic uptake route via carrier-mediated transport (Patel et al., 2012).

Until now, no histological study regarding the three different RFVTs in comparable *in vitro* and *in situ* situations was performed to assess the suitability of RFVTs as novel tumor targets. Therefore, the goal of this study was the systematic characterization of the RFVT expression pattern in both, healthy (skin, breast, aorta, kidney) and tumor tissues (SCC, melanoma, luminal A breast cancer), as well as in tissue-related *in vitro* models (A431, HUVEC, and HK2) regarding their representativeness and clinical relevance.

## Materials and Methods

### Pathohistological Staining of RFVT Expression on Human Tissue Specimen

Human biopsies of SCC, luminal A breast cancer, melanoma, aorta, and kidney were kindly provided by the Institute of Pathology at the University Hospital RWTH Aachen. This study was carried out in accordance with the recommendations of the ethics committee of the Medical Faculty of the RWTH Aachen University (Germany) with written informed consent from all subjects. All subjects gave written informed consent in accordance with the Declaration of Helsinki. The protocol was approved by the ethics committee of the Medical Faculty of the RWTH Aachen University.

Paraffin sections of 5 μm thickness were deparaffinated and incubated in sodium citrate buffer for 15 min for antigen retrieval. Sections were blocked for 1 h at room temperature (RT) using Vectastain Quickkit (Vectorlabs). Subsequently, the sections were stained for RFVT1, 2, and 3 using anti-RFVT1 (PEVR2, Assaybiotech, Fremont, CA, United States), anti-RFVT2 (PEVR1, Assaybiotech, Fremont, CA, United States), and anti-RFVT3 (C20orf54, Life Span Bioscience, Seattle, WA, United States) primary antibodies (1:100 in PBS) for 1 h at RT. The sections were then washed using PBS prior to the incubation with Alexa Fluor Plus 488-labeled anti-rabbit IgG secondary antibody (Thermo Fisher, Waltham, MA, United States) for 1 h at RT. In a final step, the tissue sections were washed with PBS and subsequently incubated with DAPI (1:5000 in PBS) for 10 min in order to visualize the cell nuclei. Images were recorded using epifluorescence microscopy (Leica DM 5500 B). Mean fluorescence intensity of the images were quantified using ImageJ. Data was normalized by background subtraction of the isotope control staining.

### Cell Lines and Cell Culture

Cells were cultured in 75 cm^2^ cell culture flasks (Cellstar^®^/TPP^®^) at 37°C, 5% CO_2_, and split when confluent using Trypsin/EDTA solution (PAN^TM^BIOTHECH). Medium was renewed three times a week. Each cell line was treated with its recommended medium, according to Table 1.

**Table 1:** Media and supplements.

Cell line	Medium
**Human umbilical vein endothelial cells** (HUVEC, Promocell^®^, Heidelberg, Germany)	**Endothelial cell growth medium 2** (Promocell^®^) 10% FCS and 1% penicillin–streptomycin
**Human kidney-2** (HK2, ATCC^®^, Manassas, VA, United States)	**DMEM/F-12** (1:1) (1X) GlutaMAX^TM^ medium (gibco^®^bylife technologies^TM^) 5% FCS and 1% penicillin–streptomycin
**A431** (Cell Lines Service, Eppelheim, Germany)	**RPMI medium** 1640 (1X) + GlutaMAX^TM^ (gib^®^cobylife technologies^TM^) 10% FCS and 1% penicillin–streptomycin


### Immunofluorescence Staining of RFVTs *in vitro*

250,000 cells of each cell line were seeded on 60 μ-glass bottom Petri dishes (ibidi^®^) and incubated at 37°C, 5% CO_2_ for 24 h, allowing the cells to adhere. To reduce endocytic uptake of the cell membrane marker, the cells were placed on ice while incubating with wheat germ agglutinin Alexa Fluor 594 (WGA-594, 1:1000 in PBS) for 30 min. Subsequently, cells were washed three times using ice cold PBS and fixed at 4°C in 4% paraformaldehyde (PFA) for 30 min. The cells were further permeabilized using 0.2% Tween-20 (Sigma Aldrich, St. Louis, MO, United States) for 15 min at 37°C and subsequently washed with PBS. To prevent unspecific binding, cells were blocked with 2% BSA in PBS for 1 h and then treated overnight with the primary antibodies (pAB) for RFVT1, RFVT2, and RFVT3 (1:100 in PBS) at 4°C. To entirely remove surplus pAB, cells were rinsed three times for 5 min using PBS prior to incubation with the secondary antibody (anti-rabbit Alexa Fluor Plus 488, Thermo Fisher, Waltham, MA, United States), diluted 1:200 in PBS for 1 h at RT. Our control samples were incubated with only the secondary antibody to exclude unspecific binding. In a final step, the cells were washed using PBS before 10 min incubation with DAPI (1:5000 in PBS) for nucleus staining. Mean fluorescence intensity of the images were quantified using ImageJ (Table 2). Data were normalized by background subtraction of the isotope control staining.

**Table 2:** Mean fluorescence intensity quantification of RFVT1, 2, and 3 in A431, HK2, and HUVEC in artificial units (AU).

	RFVT1	RFVT2	RFVT3	Control
A431	41 ± 11 AU	101 ± 14 AU	81 ± 17 AU	1 ± 1 AU
HK2	16 ± 4 AU	32 ± 8 AU	24 ± 4 AU	0 ± 1 AU
HUVEC	23 ± 5 AU	62 ± 22 AU	23 ± 4 AU	1 ± 1 AU


### Riboflavin Co-localization Experiment

Cells were cultured and seeded as previously described. All samples were incubated in its recommended medium (Table 1) containing an excess of riboflavin (1 nmol/μl, 0.5% DMSO). For co-localization with endocytic compartments, cells were additionally treated with Transferrin-AF488 (Jackson ImmunoResearch, Cambridgeshire, United Kingdom) (1:1000 in PBS). Alternatively, a 45 min incubation with MitoTracker^TM^ Deep Red (Thermo Fisher, Waltham, MA, United States) (1:1000 in PBS) was performed to assess co-localization with mitochondria. In both settings, the tracer uptake was stopped at different time points by washing three times with ice cold PBS and fixation with 4% PFA for 30 min at 4°C. Prior to staining, the cells were permeabilized using 0.1% Triton-X 100 in PBS. In order to visualize intracellular riboflavin, the samples were subsequently treated with an anti-riboflavin antibody (PAD054Ge01, Cloud-Clone, Houston, TX, United States) (1:10 in PBS) overnight at 4°C. The secondary antibody (anti-rabbit Alexa Fluor Plus 555, Thermo Fisher, Waltham, MA, United States) was added to the cells (1:200 in PBS) at RT for 1 h. Prior to confocal microscopy imaging, the cells were stained with DAPI (1:5000 in PBS) for 10 min.

### Temperature Dependence of Riboflavin Uptake

Cells were seeded as described previously. A431 cells were incubated for 2 h in the aforementioned medium containing an excess of riboflavin (1 nmol/μl). Samples were incubated at 4°C in precooled medium either on ice or at 37°C. After incubation, samples were rinsed three times with ice cold PBS before fixation with 4% PFA for 30 min. Riboflavin was stained as previously described. Nuclear staining was performed using DAPI (1:5000 in PBS) for 10 min.

### Confocal Microscopy

Confocal microscopy images were acquired using a Zeiss LSM 710 inverted confocal laser scanning microscope (Zeiss Plan-Apochromat 20x/0.8 M27, working distance = 0.55 mm). DAPI, Alexa Fluor Plus 488, Alexa Fluor 555 and MitoTracker^TM^ Deed Red were excited using the according laser lines (405, 488, and 561 nm) at laser power of 2–3%. Images were analyzed using the ImageJ JACoP (Just Another Co-localization Plugin), and the co-localization ratio (Mander’s Coefficient) was determined (Dunn et al., 2011).

### Real-Time PCR

For RNA isolation, cells were grown on 6 well plates until confluent. After washing with PBS, cells were lysed using 350 μl RLT buffer with mercaptoethanol and RNA was isolated using the Rneasy micro kit (Qiagen, Hilden, Germany). For the reverse transcriptase reaction, 1 μl oligo dT primer (100 μM) was added to 500 ng mRNA and incubated at 70°C for 5 min. Samples were placed on ice until RT was reached. Afterward, the samples were incubated with Master Mix (Qiagen, Hilden, Germany) for 1 h at 37°C. 10 ng cDNA were diluted in 3 μl water for real-time PCR. 7 μl of Master Mix were added to each sample. Quantitative real-time PCR was performed using PowerUp SYBR Green Master Mix (Thermo Fisher, Waltham, MA, United States) and ViiA^TM^ 7 Real-Time PCR System (Thermo Fisher, Waltham, MA, United States) targeting the genes of RFVT1, 2, and 3 (primer sequences are provided in Supplementary Table 1).

### Two-Photon Microscopy

Three-dimensional cell imaging was performed using an Olympus FV1000MPE multiphoton system (Mai Tai DeepSee pulsed Ti:Sapphire laser at an excitation wavelength of 800 nm and an 25x water dipping objective, pulse width = 100 femtoseconds, working distance = 2 mm, numerical aperture = 1.05). Two internal photon multipliers and their filters were adjusted to detect WGA-594 at the corresponding spectrum of 590–620 nm and Alexa Fluor Plus 488 at 490–540 nm. Image stacks were recorded at 1 μm step size. Images were analyzed using Imaris 9.2 (Bitplane).

### Statistical Analysis

Mean fluorescence quantification (*n* = 3), PCR quantification (*n* = 3) and co-localization data (*n* = 3) were statistically analyzed using the Mann–Whitney test and *p* < 0.05 was considered significant. Bonferroni correction was applied for multiple group comparisons. Error bars shown on graphs are the standard deviation (SD). Statistical analysis was performed using Graph Prism 7.0 (GraphPad Software).

## Results

### Histopathological Staining

Based on histological stainings of healthy and cancerous tissues, the potential suitability of riboflavin transporters as clinical tumor biomarkers was investigated. In this context, we selected cancer tissues where enhanced riboflavin uptake was reported. In detail, these are SCC, skin melanoma, and luminal A breast cancer. In addition to healthy skin and breast tissue, which represent the major growth sites of the selected tumors, aorta was included as a tissue strongly exposed to targeted drugs, and kidney as the main elimination organ and regulatory site for mineral, ion, and vitamin homeostasis.

**FIGURE 1:**
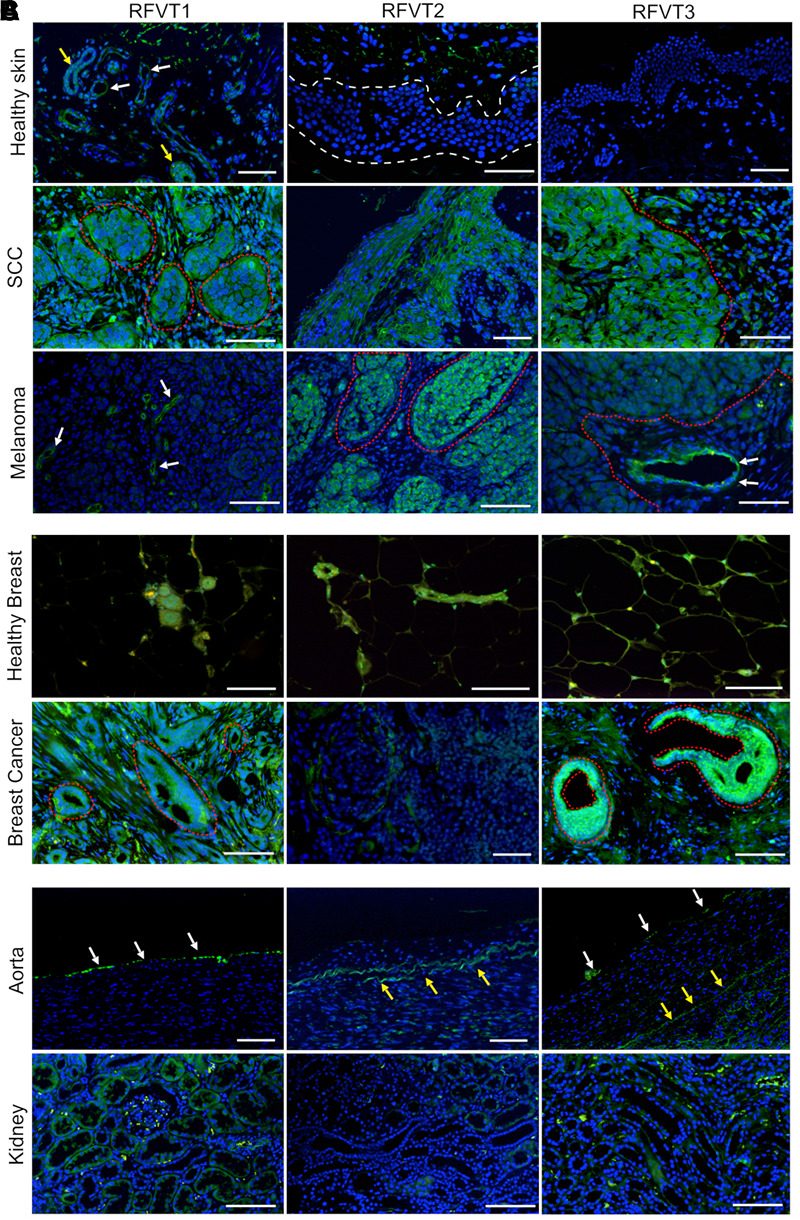
Histopathological stainings of RFVT1, 2, and 3 on SCC, skin melanoma, luminal A breast cancer and healthy control tissues (skin, breast, aorta, and kidney). RFVTs: green, nuclei: blue; scale bar: 50 μm. The tumor tissues show significant upregulation of RFVTs compared to their respective healthy organ tissues. **(A)** Healthy skin shows low expression levels of all RFVTs, with RFVT1 expression in blood vessels (white arrows) and sweat glands (yellow arrows), while lacking epithelial expression (white dotted line). In contrast, SCC presents with an elevated expression of RFVT1, 2, and 3 in cancer clusters (red dotted line). Melanoma tissues upregulate RFVT2 and 3 (red dotted line), however, only a low expression level of RFVT1 is found in the tumor vasculature (white arrows). **(B)** Healthy breast tissue shows low expression levels of RFVT1, 2, and 3 in lactiferous ducts. Luminal A breast cancer tissue is weakly positive for RFVT2 and presents significant overexpression of RFVT1 and RFVT3 in ductal carcinoma *in situ* (red dotted line). **(C)** The healthy aorta shows endothelial presence of mainly RFVT1 with low expression of RFVT3 (white arrows). Yellow arrows point to autofluorescence of elastin. Healthy kidneys have a low constitutive expression of all three RFVTs.

Healthy control skin did not display significant expression of any RFVT on the epithelium (Figure 1A), however, blood vessels and sweat glands presented with elevated expression of RFVT1. In contrast, SCC cell clusters in the subepithelial layer were highly positive for all three RFVTs. While RFVT1 and 3 were distributed intracellularly and at the cellular membranes, RFVT2 was predominantly present at the cellular surfaces (Figure 1A). Skin melanoma strongly overexpressed RFVT2 and 3, located both intracellularly and at the membranes. However, skin melanoma showed only low expression of RFVT1 (Figure 1A), indicating tumor type specific upregulation of individual RFVTs. In healthy breast tissue (Figure 1B), positive staining for all RFVTs was limited to the lactiferous ducts. In contrast, in luminal A breast cancer (Figure 1B), we found a strongly enhanced intracellular and membranous presence of RFVT1 and RFVT3, while RFVT2 was only slightly overexpressed.

In comparison to the vascular expression of all three RFVTs in tumors, mainly RFVT1 was present on blood vessels of the healthy skin as well as the endothelia of the aorta. Furthermore, healthy kidney tissues presented with low expression levels of all three RFVTs in the proximal and distal tubules. Of the three RFVTs, RFVT1 expression was higher than that of RFVT2 and 3 (Figure 1C).

**FIGURE 2:**
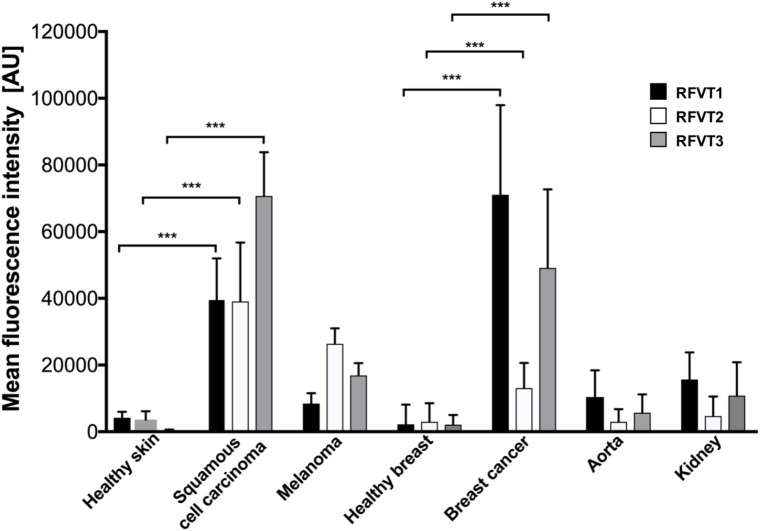
Mean fluorescence intensity quantification of histopathological staining of RFVT1, 2, and 3 in SCC, skin melanoma, luminal A breast cancer, and healthy control tissues (skin, breast, aorta, and kidney). All tumors significantly overexpress all three RFVTs compared to their respective healthy skin and breast tissues. Each tumor type presents an individual RFVT expression pattern showing the highest expression of RFVT1 in breast cancer, the highest RFVT2 expression in melanoma and the highest RFVT3 expression in SCC. Healthy aorta and kidneys significantly overexpress RFVT1 and 3 compared to healthy skin and breast tissue. Mean fluorescence intensity was quantified using ImageJ in artificial units (AU). Error bars represent standard deviation; ^∗∗∗^*p* < 0.0001, *n* = 3. Data was normalized using background subtraction of the isotype control.

Mean fluorescence intensity quantification of the histopathological images revealed a 9-fold higher RFVT1, an 11-fold higher RFVT2, and a 187-fold higher RFVT3 signal in SCC tissues compared to healthy skin. Similar tendencies were observed for the melanoma (2-fold for RFVT1, 7-fold for RFVT2, and 44-fold for RFVT3) and luminal A breast cancer tissues (31-fold for RFVT1, 4-fold for RFVT2, and 22-fold for RFVT3) in comparison with their respective healthy tissue (Figure 2). It is important to note, that there were significant differences in RFVT expression levels between the healthy samples. For example, healthy kidneys showed a 7-fold higher level of RFVT1 and 5-fold higher level of RFVT3 compared to healthy breast tissue, indicating that side effects of riboflavin-targeted therapeutics need to be carefully excluded for this organ. Nevertheless, fluorescence intensities were still 7-fold higher in SCC, 1.6-fold higher in melanoma, and 5-fold higher in luminal A breast cancer than in the kidney.

### RFVT Expression Analysis *in vitro*

Based on the histopathological analysis of patient biopsies, we identified a low level RFVT expression in healthy tissues and the overexpression of RFVTs in all cancerous tissues. We selected cell lines that are representative for SCC (A431 cells), as well as endothelial (HUVEC) and kidney epithelial cells (HK2), due to their strong exposure to riboflavin.

**FIGURE 3:**
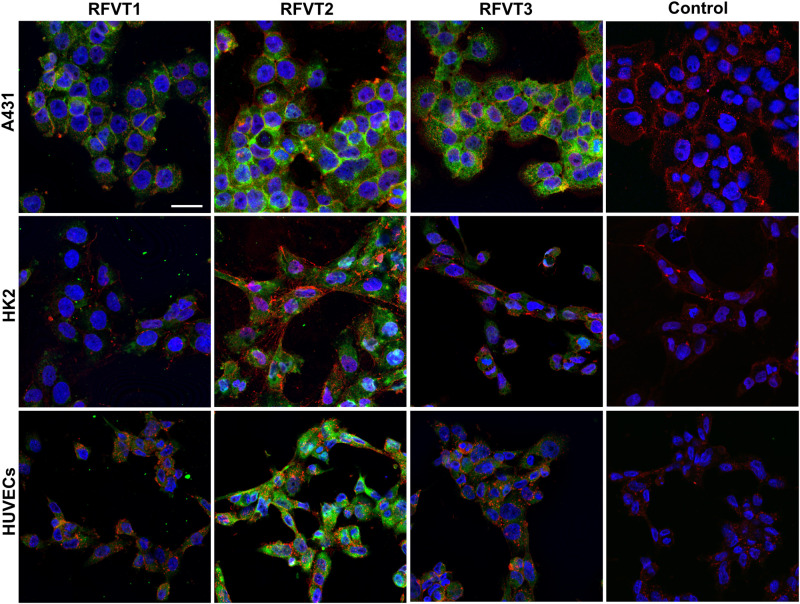
*In vitro* immunofluorescence stainings of RFTV1, 2, and 3 in A431, HK2, and HUVEC. RFVTs: green, cell membrane: red, nuclei: blue; scale bar: 30 μm. A431 cells strongly upregulate all three RFVTs, both intracellularly and on the cell membrane. HK2 cells present low expression levels of RFVT1 and 3, while RFVT2 is moderately present. HUVEC present strong expression of RFVT2 and weak RFVT1 and 2 expression. Isotype IgG antibodies were used for control samples. *n* = 3.

In immunofluorescence images of A431 cells, representing human SCC tissue, a strong upregulation of all three RFVTs (Figure 3 and Table 2) was observed, distributed both intracellularly and on the cell membrane, pointing to its potential surface availability. While in HK2 cells only low expression levels of all three RFVTs were found both intracellularly and on the cell membrane, HUVEC presented low level expression for RFVT1 and 3, but similar to A431 cells, the overexpression of RFVT2 (Table 2). This may be due to the fact that HUVEC are immature embryonal endothelial cells with strong phenotypic similarities to the tumor endothelium.

### Quantification of RFVT1, 2, and 3 Genomic Expressions and Membrane Availability

Following up on the histological findings for our cell lines, characterization of RFVT expression was carried out at the genomic and protein level by means of qPCR and three-dimensional two-photon microscopy, respectively.

**FIGURE 4:**
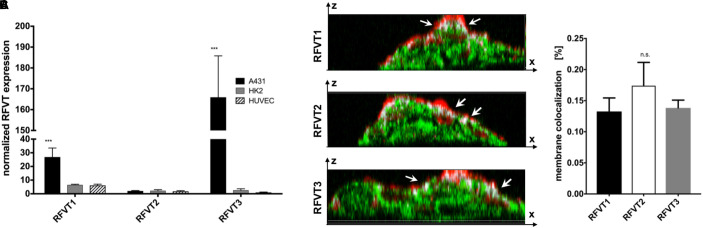
Quantitative analysis of RFVT1, 2, and 3 mRNA expression and cellular localization. **(A)** The quantitative PCR analysis of RFVT mRNA expression in all three selected cell lines (A431, HK2, and HUVEC) confirmed histological findings, showing significantly elevated mRNA levels for RFVT1 and 3 in A431 cells. In contrast, HK2 and HUVEC presented low mRNA levels for all three RFVTs (^∗∗∗^*p* < 0.0001, *n* = 3). **(B)** Z-stack confocal microscopy images of RFVT stainings in A431 cells show the co-localization (white, indicated by arrows) between the apical cell membrane (red) and the RFVTs (green). Scale bar = 15 μm. **(C)** Three-dimensional volume co-localization analysis and determination of the Mander’s Coefficients for RFVT colocalization with the cell membrane in A431 cells was performed with the IMARIS co-localization plugin. No significant differences between the membrane presences of the three RFVTs were found (n.s., not significant).

In line with our histological stainings, the mRNA levels of RFVT1 and RFVT3 in A431 were the highest amongst the tested cells. In detail, A431 cells showed an mRNA level of RFVT1 being four times higher than in HK2 cells and HUVEC (*p* = 0.0034), and the mRNA level for RFTV3 was 63-fold higher (*p* < 0.0001) (Figure 4A). No significant differences in mRNA levels could be detected between HK2 and HUVEC for RFVT1 and RFVT3 and between all three cell types regarding RFVT2, indicating constitutive presence of this RFVT.

The membrane availability and cellular localization of the RFVTs were investigated by three-dimensional two-photon microscopy using the cell line with the highest RFVT expression. Z-stack images of A431 cells were recorded, displaying its intracellular localization as well as its co-localization with the wheat germ agglutinin-labeled cell membrane. Our data suggest that all three RFTVs are partially present on the apical cell membrane and also distributed intracellularly (Figure 4B). Volume co-localization estimations based on the Mander’s Coefficient revealed a 13.2–17.4% co-localization of the total RFVT signal with our membrane staining, without significant differences between the three RFVTs (Figure 4C). Consequently, all three RFVTs are surface accessible.

### Cellular Internalization and Trafficking of Riboflavin

The RFVT expression pattern of A431 and HK2 cells presented with a high homology to SCC and healthy kidney tissue, respectively, and therefore these cell lines can be considered to represent suitable *in vitro* models. For that reason, cellular uptake and compartmental co-localization of riboflavin were investigated in these two cell lines.

The uptake kinetics and pathway of riboflavin internalization in cancer cells compared to healthy cells was determined by co-localization studies using the well-established endocytosis marker transferrin. The readout parameters for co-localization were the Mander’s Coefficients, M1 and M2. M1 is the overlap ratio of total riboflavin with transferrin. It represents the percentage of total intracellular riboflavin following the receptor-mediated endocytosis pathway of transferrin. Reversely, M2 is the overlap ratio of total transferrin with riboflavin, an indicator for endosomal saturation with intracellular riboflavin.

**FIGURE 5:**
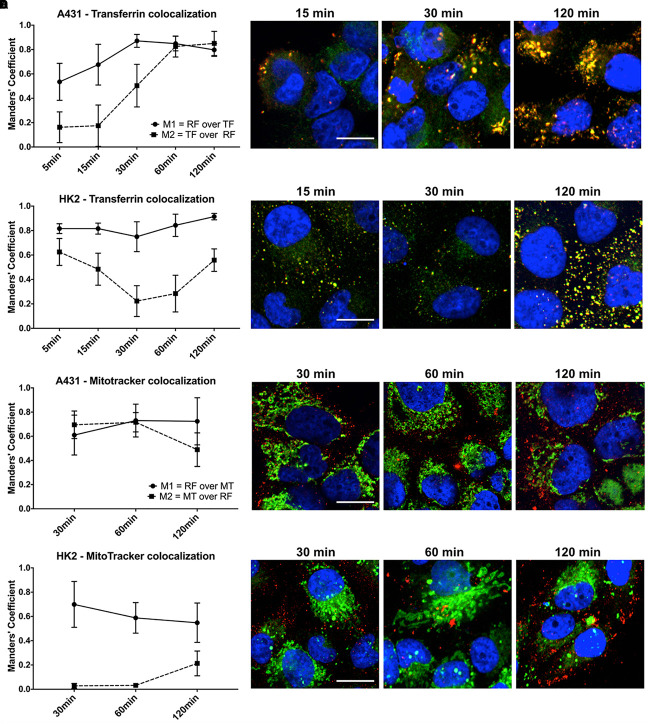
Quantification of riboflavin and transferrin co-localization in A431 and HK2 cells based on confocal microscopy and Mander’s Coefficient. Cells were incubated with riboflavin (red) and either Transferrin-AF488 (green) or MitoTracker^TM^ Deep Red (green) for 120 min. Nuclear staining (blue) was achieved with DAPI. Co-localization (yellow) between riboflavin and transferrin or MitoTracker^TM^ Deep Red was quantified using Mander’s Coefficients with the ImageJ plugin JACoP. M1 = the overlap ratio of total riboflavin with transferrin or MitoTracker^TM^ Deep Red, M2 = the overlap ratio of total transferrin or MitoTracker^TM^ Deep Red with riboflavin. Scale bar = 10 μm. Riboflavin colocalized strongly with transferrin in both **(A)** A431 cells as well as **(B)** HK2 cells, whereas transferrin only moderately colocalized with riboflavin in HK2 cells. Similarly, riboflavin strongly colocalized with MitoTracker^TM^ Deep Red in both **(C)** A431 cells and **(D)** HK2 cells. In contrast, MitoTracker^TM^ Deep Red showed low colocalization with riboflavin.

The uptake of riboflavin was strongly linked to the endocytosis of transferrin. Already after 5 min of incubation with riboflavin and transferrin in A431 cells 53.5% of total riboflavin was co-localized with transferrin (M1), increasing linearly to 87.1% after 30 min (*p* < 0.0001). M1 remained over 80% at later time points, suggesting receptor-mediated endocytosis as the primary uptake mechanism (Figure 5A). Similarly, in HK2 cells the majority of internalized riboflavin was following the endocytic pathway of transferrin, reflected by an over 80% overlap of total intracellular riboflavin with transferrin.

Moreover, based on confocal images, the intracellular riboflavin accumulation in A431 cells was significantly higher than in HK2 cells. This was further substantiated by the differences for M2 between A431 and HK2 cells. While in A431 cells, M2 reached approximately 80% after 60 min, indicating efficient riboflavin uptake and saturation of most transferrin-filled compartments, M2 for HK2 cells dropped significantly within the first 60 min of co-incubation from 62 to 22% (*p* < 0.0001). With increasing transferrin internalization over time, less transferrin-labeled compartments in HK2 cells were filled with riboflavin, due to the low riboflavin uptake (Figure 5B).

### Confocal Microscopy and Quantification of Riboflavin and MitoTracker^TM^ Deep Red Co-localization in A431 and HK2

In addition to the uptake mechanism of riboflavin, we investigated its specific intracellular translocation route. Riboflavin, as a precursor to FMN and FAD, is associated with mitochondrial energy metabolism. Therefore, we performed co-localization studies in combination with MitoTracker^TM^ Deep Red, a marker for mitochondria, to obtain additional information about the sub-cellular localization of riboflavin. Overall, in both A431 and HK2 cells, the overlap ratio between total intracellular riboflavin and MitoTracker^TM^ (M1) was above 50% at all time points, changing only slightly within 120 min from 61 to 72% (*p* < 0.0001) in A431 (Figure 5C) and dropping from 69 to 55% in HK2 cells (*p* < 0.0001) (Figure 5D). This indicates that riboflavin is intracellularly translocated through the mitochondria in both cell lines. Similar to the previous co-localization study, M2 (overlap ratio between MitoTracker^TM^ and intracellular riboflavin) pointed to major differences in the riboflavin uptake kinetics between A431 and HK2 cells. While after 30 min of riboflavin internalization, M2 was at 72% in A431, M2 in HK2 cells was only at 3.2%. This demonstrates that in HK2 cells only a very small fraction of mitochondria was co-localized, and thus, saturated with riboflavin.

### ATP Dependency of Riboflavin Uptake

After a 2 h incubation at 37°C, we observed elevated levels of riboflavin within A431 cells in the form of weak hazy fluorescence signals, as well as dot-shaped compartmental accumulation. After incubation of the cells at 4°C hardly any intracellular riboflavin distribution was found, and the majority of the staining was present the cell membrane (Supplementary Figure 1), pointing to an ATP-dependent uptake mechanism.

## Discussion

In this study, we investigated the riboflavin transporter (RFVT) expression patterns of cancerous and healthy tissues and demonstrated the tumor-specific overexpression of RFVTs in SCC, melanoma and luminal A breast cancer tissues. With considerably lower constitutive levels of RFVTs in healthy tissues, the strongest RFVT overexpression was observed for RFVT3 in SCC, with a 186-fold increase in mean fluorescence intensity compared to healthy skin. Similar to SCC, we also found a 42-fold higher RFVT3 expression in melanoma as well as a 22-fold higher expression in luminal A breast cancer tissue compared to their corresponding healthy counterpart. A high tumor-specific RFVT3 expression as well as its important role in tumor metabolism was previously described in esophageal squamous carcinoma (ESCC) (Jiang et al., 2014) and glioma (Fu et al., 2016). In detail, RFVT3 silencing in glioma and ESCC cells inhibited cell proliferation and augmented apoptosis via decreased anti-apoptotic proteins and cell cycle arrest. Additionally, impaired mitochondrial function going along with reduced cellular ATP levels were reported for RFVT3 silenced ESCC cell lines and hampered migration and invasion were detected in RFVT3 silenced glioma cells (Jiang et al., 2014; Fu et al., 2016). Reversely, the overexpression of RFVT3 in ESCC cell lines led to enhanced cell proliferation, higher levels of anti-apoptotic proteins, improved cisplatin resistance and increased tumorgenicity (Jiang et al., 2014; Long et al., 2018). Interestingly, RFVT3 expression increased with WHO grade of glioma (Fu et al., 2016) and throughout development of ESCC (Long et al., 2018), pointing to a prognostic relevance. While RFVT3 seems pivotal for tumor survival and growth, less is known about the tumor-associated functions of RFVT1 and 2. Apart from significant upregulation of RFVT3, the three tumor types investigated in this study presented individual RFVT expression patterns, with RFVT3 being the predominant transporter in SCC, RFVT2 in melanoma, and RFVT1 in luminal A breast tumor tissue. The tissue- and stage-specific tumor marker expression is not uncommon and could be used for the development of novel diagnostic and therapeutic approaches (Vaidyanathan and Vasudevan, 2012; Nagpal et al., 2016). Since all tumors showed simultaneous upregulation of more than one RFVT, dual- or triple-targeted drug formulations may be ideal to increase tumor uptake. However, further studies are required to elucidate the potential diagnostic, prognostic, and therapeutic value of all RFVTs.

Nonetheless, it is important to consider RFVT expression in healthy tissues, particularly in those that would be strongly exposed to riboflavin-targeted therapeutics. For example, RFVT expression in sweat glands of the skin may lead and neutrophilic eccrine hidradenitis (Wu and Krishnamurthy, 2018) after the administration of RFVT-targeted therapeutics. Another organ that may potentially be affected by RFVT-targeted drugs is the kidney, which is known to modulate the body’s vitamin homeostasis: the renal tubular recovery of essential vitamins in the proximal tubules has been described for vitamin B12 (Birn, 2006), B9 (Birn et al., 2005), (Castro et al., 2008), as well as riboflavin (Kumar et al., 1998; Yanagawa et al., 2000). Thus, it is not surprising, that healthy kidneys showed strong presence of RFVTs, with 5- to 7-fold higher RFVT1 and RFVT3 levels in the proximal tubules compared to healthy breast or skin tissues. Similarly, endothelial cells of both healthy and tumor vasculature would represent highly exposed sites of riboflavin-targeted therapeutics (Nichols and Bae, 2012). While in the aorta only a low constitutional expression of RFVT1 and 3 was found, tumor endothelial cells strongly expressed all three RFVTs. Due to the non-exclusivity of RFVT expression on tumor cells, similar to other prominent tumor targets, such as the somatostatin receptors expressed in lymphatic and neuroendocrine tissue, kidneys, and prostate (Reubi et al., 1997), or the folate receptor being expressed in healthy kidneys (Parker et al., 2005), the offsite accumulation of riboflavin-targeted therapeutics needs to be carefully evaluated concerning the risk-benefit trade-off between efficacy and toxicity (Pignatti et al., 2015). Even though toxicity to healthy tissues cannot be fully avoided, it can be partially mitigated by the tumor marker selection and combination. Of the three cancer types investigated, based on the overall RFVT availability, luminal A breast cancer and SCC appear highly suitable for RFVT targeting. Luminal A breast cancer presented the highest RFVT1 and second highest RFVT3 expression, and the overexpression ratios compared to healthy kidney were about 4.5-fold for both RFVTs. SCC can be considered to be even more suited for RFVT targeting, displaying the highest RFVT2 and 3 levels amongst the tested tumors and an 8-fold higher RFVT2 level compared to kidney as well as a 13-fold higher level compared to aorta. For RFVT3 a 7- and 12-fold higher expression was found, respectively. Despite the higher overexpression of RFVT3, RFVT2 might clinically also be a highly interesting target due to the better SCC to kidney expression ratio and the thus, expectable low toxicity to this organ of risk. Moreover, studies have shown RFVT2 to have the highest affinity toward native riboflavin, while RFVT3 has been reported to mediate the highest riboflavin uptake velocity (Yao et al., 2010). When choosing the ideal RFVT targeting concept these aspects also need to be considered, and further studies are required to elucidate which RFVT receptor or combination of receptors should ideally be targeted to maximize drug delivery to tumors but avoiding toxicity the healthy tissues. Compared to healthy kidney and aorta, melanoma neither shows significant overexpression of RFVT1 nor 3, however RFVT2 levels are increased by 5.5- and 9-fold, respectively. This demonstrates that different tumor types selectively overexpress distinct RFVT receptors. Thus, if not an unselective RFVT targeting concept is chosen, the RFVT expression pattern of each tumor entity needs to be carefully studied before a targeted drug or diagnostic agent is developed. In addition, besides the careful target selection, it is noteworthy that potential side effects from RFVT targeting can be minimized by using drug delivery systems that hardly reach extravascular targets due to their large size and the low “Enhanced Permeability and Retention” (EPR) effect in most healthy organs (Kobayashi et al., 2013; Kobayashi and Choyke, 2016).

In addition to the histopathological characterization of RFVT expression on human biopsies, we identified A431 cells as an adequate *in vitro* model for studies involving riboflavin and riboflavin transporter targeting. All three RFVTs, were strongly expressed in A431 cells as well as in SCC. The RFVTs were presented at the apical membrane promoting a fast and efficient uptake of riboflavin. This is a highly desired feature of tumor-targeted pharmaceuticals providing an explanation for the promising results obtained with riboflavin-targeted diagnostics (Tsvetkova et al., 2016, 2017; Beztsinna et al., 2017; Pal et al., 2017) and therapeutics (Bareford et al., 2013; Beztsinna et al., 2016) at the *in vitro* and *in vivo* level.

Cellular uptake mechanisms of vitamins are manifold and complex and can involve, in the case of folate, functionally different transporters and uptake mechanisms such as receptor-mediated endocytosis (Wibowo et al., 2013), antiporter (Desmoulin et al., 2012), and passive diffusion (Zhao et al., 2009). Especially for theranostic applications and nano-medicines, receptor-mediated endocytosis is often a prerequisite, since larger drug delivery systems usually are not capable of passing the narrow carrier channels (Sahay et al., 2010). However, receptor-mediated endocytosis covers various mechanisms, such as clathrin-mediated or caveolae-mediated endocytosis, micropinocytosis or phagocytosis, determining the intracellular translocation of its cargo (Doherty and McMahon, 2009; Treuel et al., 2013). One extensively studied mechanism is the clathrin-dependent endocytosis of transferrin (Mayle et al., 2012). In consensus with previous studies (Bareford et al., 2008, 2013), we suggest clathrin-mediated endocytosis as the primary cellular uptake mechanism of riboflavin in A431 cells, due to strong endosomal co-localization with transferrin, comprising up to 80% of the total amount of internalized riboflavin. Nevertheless, without studies using endocytic inhibitors, the impact of a secondary active transport of riboflavin via membrane carriers cannot be determined for the non-co-localized riboflavin. Furthermore, ATP depletion did not completely inhibit intracellular accumulation of riboflavin, suggesting passive diffusion as another alternative internalization route. However, this pathway was observed in particular during riboflavin over-supplementation *in vitro* and does not pose a suitable option for efficient drug delivery (Kumar et al., 1998). Not only did our data show that riboflavin is mainly taken up by endocytosis, riboflavin is also translocated to a high degree to the mitochondria, a favorable compartment for cytotoxic drug delivery (Weinberg and Chandel, 2015). As riboflavin is involved in the cellular respiratory chain (Mansoorabadi et al., 2007) and its deficiency is associated with mitochondrial dysfunction (Marashly and Bohlega, 2017; Ozsvari et al., 2017; Udhayabanu et al., 2017), it may be highly suited to direct tumor therapeutics specifically into the mitochondria. However, it is not yet clear which receptor predominantly mediates endocytic riboflavin internalization and trafficking to the mitochondria.

Further confirmation of the uptake mechanism and intracellular translocation was provided in HK2 cells, the representative cell line for healthy kidney. Although these cells exhibited relatively low expression levels of all RFVTs, riboflavin mainly underwent receptor-mediated endocytosis and was directed to the mitochondria. However, due to the slow uptake of riboflavin in HK2 cells, saturation levels of transferrin-labeled endosomes and MitoTracker^TM^-labeled mitochondria with intracellular riboflavin were significantly lower than in A431 cells.

While we were able to validate A431 and HK2 as suitable *in vitro* models for SCC and healthy kidney, we ruled out HUVEC as a representative model for healthy endothelium regarding riboflavin uptake. Contradictory to the endothelial presence of RFVT1 in aorta and small blood vessels, HUVEC overexpressed RFVT2 in similar manners as A431 cells. This is likely due to the embryonal origin of HUVEC, which renders this cell line a more suitable *in vitro* model for tumor endothelia rather than healthy endothelium. Unfortunately, the representativeness of aortic endothelial cells could not be investigated, which represents a limitation of this study.

Interestingly, riboflavin is not the first vitamin implicated in active tumor targeting. Folate, as riboflavin, is an essential vitamin of the vitamin B family and has been investigated for active tumor targeting in a range of diagnostic (Meier et al., 2010; van Dam et al., 2011; Ocak et al., 2015) and therapeutical applications (Zwicke et al., 2012; Ledermann et al., 2015; Vergote and Leamon, 2015). In this context, a translationally advanced example is the folate-conjugate vinblastine for the treatment of ovarian and non-small-cell lung cancer that is currently investigated in a phase 3 clinical trial (Vergote and Leamon, 2015). Moreover, folate is internalized via both ubiquitously expressed membrane carriers (Zhao et al., 2011) and high affinity folate receptors, which are overexpressed in various cancers, promoting receptor-mediated endocytosis (Parker et al., 2005; Cheung et al., 2016). Although only limited studies regarding riboflavin internalization and intracellular processing exist due to the recent discovery of the riboflavin transporters (Moriyama, 2011), available data are encouraging showing a strong homology between folate receptors and riboflavin transporters regarding tissue expression and uptake. It is important to note that folate receptors are also strongly expressed in healthy kidneys and although there is only a 3-fold higher receptor expression in ovarian cancer (Parker et al., 2005), folate receptor-targeted drugs perform well in preclinical and clinical trials (van Dam et al., 2011; Ledermann et al., 2015; Vergote and Leamon, 2015). Thus, it is encouraging that our data revealed a 5- to 7-fold RFVT3 overexpression ratio in SCC and luminal A breast cancer compared to healthy kidneys, exceeding the folate receptor expression ratio in ovarian cancers. Thus, for these types of cancer RFVTs might be highly promising therapeutic and diagnostic targets.

## Conclusion

Our study provides a systematic and comparative immunohistological analysis of both *in vitro* and *in situ* samples from multiple cancer types and high-risk healthy tissues, in order to assess the potential risks and benefits of targeting RFVT isomers for clinical translational applications. We identified the heterogeneous overexpression patterns of riboflavin transporters in clinical cancer samples and demonstrated its potential for tumor targeted drug delivery. We strongly encourage the readers of this article to put effort in further investigations regarding the individual function of each RFVT and the development of diagnostics and targeted drugs addressing the riboflavin uptake mechanisms.

## Author Contributions

ZW and FK contributed to conception, design of the study, and corrected the manuscript. LB collected the experimental data and wrote the manuscript. ZW, MvZ, LB, DS, SvS, MS, and SA-R contributed to data analysis and experimental execution. All authors contributed to manuscript revision, read and approved the submitted version.

## Conflict of Interest Statement

The authors declare that the research was conducted in the absence of any commercial or financial relationships that could be construed as a potential conflict of interest.
