# Corrigendum: Evaluation of Riboflavin Transporters as Targets for Drug Delivery and Theranostics

**DOI:** 10.3389/fphar.2020.617329

**Published:** 2020-11-26

**Authors:** Lisa Bartmann, David Schumacher, Saskia von Stillfried, Marieke Sternkopf, Setareh Alampour-Rajabi, Marc A. M. J. van Zandvoort, Fabian Kiessling, Zhuojun Wu

**Affiliations:** ^1^ Institute for Experimental Molecular Imaging, University Clinic, RWTH Aachen University, Aachen, Germany; ^2^ Institute for Molecular Cardiovascular Research, University Clinic, RWTH Aachen University, Aachen, Germany; ^3^ Institute of Pathology, University Clinic, RWTH Aachen University, Aachen, Germany; ^4^ Department of Genetics and Molecular Cell Biology, School for Cardiovascular Diseases (CARIM), School for Oncology and Developmental Biology (GROW), Maastricht University, Maastricht, Netherlands

**Keywords:** riboflavin, RFVT, theranostic, nanocarrier, cancer therapy, targeting molecules

In the original article, there was an error. A statement on author contribution was incorrect. A correction has been made to the **Author Contributions**:

## Author Contributions

ZW and FK contributed to conception, design of the study, and corrected the manuscript. LB collected the experimental data and wrote the manuscript. ZW, MvZ, LB, DS, SvS, MS, and SA-R contributed to data analysis and experimental execution. All authors contributed to manuscript revision, read and approved the submitted version.

The authors apologize for this error and state that this does not change the scientific conclusions of the article in any way. The original article has been updated.

