# Corrigendum: The Protective Effects of Lipid-Lowering Agents on Cardiovascular Disease and Mortality in Maintenance Dialysis Patients: Propensity Score Analysis of a Population-Based Cohort Study

**DOI:** 10.3389/fphar.2022.894462

**Published:** 2022-03-31

**Authors:** Ming-Hsien Tsai, Mingchih Chen, Yen-Chun Huang, Hung-Hsiang Liou, Yu-Wei Fang

**Affiliations:** ^1^ Division of Nephrology, Department of Internal Medicine, Shin-Kong Wu Ho-Su Memorial Hospital, Taipei City, Taiwan; ^2^ Department of Medicine, School of Medicine, Fu Jen Catholic University, New Taipei City, Taiwan; ^3^ Graduate Institute of Business Administration, College of Management, Fu Jen Catholic University, New Taipei City, Taiwan; ^4^ AI Development Center, Fu Jen Catholic University, New Taipei City, Taiwan; ^5^ Division of Nephrology, Department of Internal Medicine, Hsin-Jen Hospital, New Taipei City, Taiwan

**Keywords:** dialysis, National Health Insurance Research Database, lipid-lowering agents, mortality, major adverse cardiovascular events

In the published article, Mingchih Chen was incorrectly included as a corresponding author. The only corresponding author should be Yu-Wei Fang.

The authors apologize for this error and state that this does not change the scientific conclusions of the article in any way. The original article has been updated.

