# Supplementary material for: Evaluation of Riboflavin Transporters as Targets for Drug Delivery and Theranostics
Source: Front Pharmacol. 2019 Feb 6;10:79. doi: 10.3389/fphar.2019.00079 (PMC6372557; doi:10.3389/fphar.2019.00079)
Supplement: Supplementary file 1 [file Data_Sheet_1.docx]

Supplementary Material

**Evaluation of Riboflavin Transporters as Targets for Drug Delivery and Theranostics**

Lisa Bartmann^1,2^, David Schumacher^2^, Saskia von Stillfried^3^, Marieke Sternkopf^2^, Setareh Alampour-Rajabi^2^, Marc van Zandvoort^2,4^, Fabian Kiessling^1^*, Zhuojun Wu^1,2*^

*** equal contributions as corresponding authors:**

Dr. rer. nat. Zhuojun Wu, Department of Experimental Molecular Imaging, University Clinic, RWTH Aachen University, Pauwelsstrasse 30, 52074 Aachen, Germany.

Email: [zwu@ukaachen.de](mailto:zwu@ukaachen.de)

Phone: +49 (0) 241 8036922

Prof. Fabian Kiessling, Department of Experimental Molecular Imaging, University Clinic, RWTH Aachen University, Pauwelsstrasse 30, 52074 Aachen, Germany.

Email: [fkiessling@ukaachen.de](mailto:f.kiessling@ukaachen.de)

Phone: +49 (0) 241 8080116

**Supplementary Table 1: Primer sequences for RFVT1, 2, 3 and GAPDH**

| RFVT1-forward: | AAAAGACCTTCCAGAGGGTTG |
| --- | --- |
| RFVT1-reverse: | AGCACCTGTACCACCTGGAT |
| RFVT2-forward: | CCTTTCCGAAGTGCCCATC |
| RFVT2-reverse: | AGAAGGTGGTGAGTAGG |
| RFVT3-forward: | CCCTGGTCCAGACCCTA |
| RFVT3-reverse: | ACACCCATGGCCAGGA |
| GAPDH-forward: | CCTGCACCACCAACTGCTTA |
| GAPDH-reverse: | GGCCATCCACAGTCTTCTGAG |


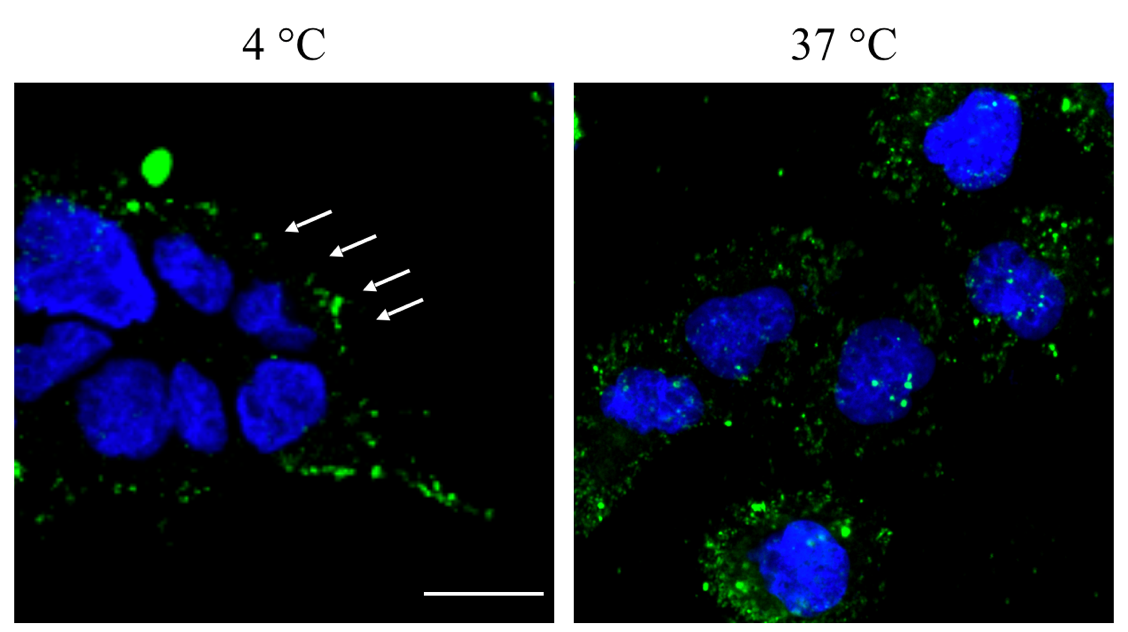


**Supplementary Figure 1. Temperature dependent uptake of riboflavin in A431 cells.** At 4°C, the majority of riboflavin (green) was located on the outer membrane of the cells (white arrows). In contrast, at 37°C riboflavin was distributed throughout the entire cell and around the nuclei (blue). Scale bar = 10 µm.
